# Os Níveis de Atividade Física Mudam ao Longo do Tempo em Indivíduos com Doença Arterial Periférica

**DOI:** 10.36660/abc.20220368

**Published:** 2022-07-07

**Authors:** Alexandre Pereira, Leandro Franzoni

**Affiliations:** 1 Universidade Federal do Rio Grande do Sul Programa de Pós-Graduação em Ciências da Saúde: Cardiologia e Ciências Cardiovasculares Porto Alegre RS Brasil Universidade Federal do Rio Grande do Sul – Programa de Pós-Graduação em Ciências da Saúde: Cardiologia e Ciências Cardiovasculares, Porto Alegre, RS – Brasil; 2 Hospital de Clínicas de Porto Alegre Ambulatório de Cirurgia Vascular Porto Alegre RS Brasil Hospital de Clínicas de Porto Alegre – Ambulatório de Cirurgia Vascular, Porto Alegre, RS – Brasil

**Keywords:** Atividade Física, Doença Arterial Periférica, Aceleração, Velocidade, Caminhada, Exercício

A atividade física desempenha um papel fundamental na melhora da capacidade funcional e da função cardiovascular na doença arterial periférica (DAP).^1^ Existe relação direta entre a melhora do consumo de oxigênio de pico (VO_2_pico) e a redução do risco de mortalidade; além disso, a melhora dos sintomas relacionados à claudicação intermitente proporciona melhor qualidade de vida (QV).^2,3^ No entanto, a avaliação do nível de atividade física é frequentemente realizada em estudos transversais, mostrando que um maior nível de atividade física está correlacionado com maior capacidade funcional, por exemplo. No entanto, os estudos não consideram a exposição em um determinado período, deixando em aberto se há alterações nos parâmetros de risco cardiovascular e níveis de atividade física nesses indivíduos após um acompanhamento. É o que Cucato et al.,^4^ analisaram nesta edição dos Arquivos Brasileiros de Cardiologia.

Inicialmente, traremos as questões metodológicas do estudo, que teve início em 2015 e incluiu, na primeira fase, 268 pacientes. Após 2 anos de seguimento, 72 pacientes foram reavaliados na segunda fase. Diferentes parâmetros de risco cardiovascular e níveis de atividade física foram avaliados usando um acelerômetro triaxial GT3X+ (Actigraph, Pensacola, FL, EUA). Aqui é importante destacar o primeiro ponto positivo, que foi o uso de um acelerômetro para controlar os níveis de atividade física, pois muitos estudos utilizam um questionário para controlar o nível de atividade física.^5^ Todos os pacientes foram orientados a usar o acelerômetro por 7 dias consecutivos, removendo-o apenas para dormir ou tomar banho. O aparelho foi fixado no lado direito do quadril e, para análise, foi necessário um mínimo de 10 horas de registro diário de atividade física. Foram considerados válidos aqueles que tiveram pelo menos 4 dias de atividade, 3 dias de semana e 1 dia de final de semana. Valorizamos muito essa parte, pois é um método simples que nos fornece informações de altíssima qualidade. Entre os diferentes parâmetros de risco cardiovascular, podemos destacar a pressão arterial, a modulação autonômica cardíaca e a rigidez arterial. Enquanto a capacidade funcional foi avaliada através do teste de caminhada de 6 minutos. Apesar de ser um teste extremamente seguro, eficaz e reprodutível, apresenta limitações, e aqui fica uma sugestão para estudos futuros que avaliem o VO_2_pico e suas variáveis por meio de um teste específico utilizando o protocolo de Gardner.^6–8^

Em relação aos resultados, podemos destacar que os pacientes reduziram seus níveis de atividade física total durante esses 2 anos (2,257 ± 774,5 min/semana pré versus 2,041 ± 676,2 min/semana pós, p = 0,001). Algo importante e preocupante é que o índice tornozelo-braquial (ITB) também foi significativamente reduzido após o seguimento de dois anos (0,62 ± 0,20 pré versus 0,54 ± 0,20, p = 0,003). Por que esse resultado é preocupante? Alguns estudos mostram que o ITB é um marcador prognóstico em indivíduos com DAP; combinando isso com a redução dos níveis de atividade física, teremos um cenário alarmante para essa população. No presente estudo, a QV não foi avaliada; entretanto, em um cenário de piora de diferentes parâmetros, podemos especular que esses indivíduos provavelmente pioraram sua QV. Do ponto de vista dos parâmetros cardiovasculares, destacamos a piora da variabilidade da frequência cardíaca medida pelo desvio padrão dos intervalos RR (p < 0,001).

Um dos pontos que chama muito a atenção é que não foi mencionado se os indivíduos da linha de base participaram de algum programa de treinamento físico, pois o grupo de pesquisa do Dr. Cucato é referência no Brasil e uma das referências no mundo em reabilitação de DAP.^9^ Muitas vezes, quando os indivíduos participam de um programa de reabilitação, aumentam seus níveis de atividade física diária. No entanto, ao final do programa, a tendência é reduzir seus níveis de atividade física.^10^ A mensagem do artigo é muito clara e nos faz pensar na importância do acompanhamento desses indivíduos ao longo do tempo. A DAP acaba sendo uma doença subdiagnosticada; ou seja, os indivíduos apresentam sintomas, mas muitas vezes não se importam, chegando a um nível crítico que gera diversas consequências negativas, como redução da capacidade funcional, piora da QV e piora dos parâmetros cardiovasculares. Novos estudos devem ser desenhados a priori para um longo acompanhamento desses indivíduos para que tenhamos mais informações sobre a evolução da doença e quais benefícios um maior nível de atividade física pode proporcionar.
