# Supplementary material for: Os Níveis de Atividade Física Mudam ao Longo do Tempo em Indivíduos com Doença Arterial Periférica
Source: Arq Bras Cardiol. 2022 Jul 7;119(1):67–8. [Article in Portuguese] doi: 10.36660/abc.20220368 (PMC9352132; doi:10.36660/abc.20220368)
Supplement: Translation

# Translation: Physical Activity Levels Change Over Time in Individuals with Peripheral Arterial Disease

**Authors:** Alexandre Pereira, Leandro Franzoni

**Affiliations:** 1 Porto Alegre RS Brazil Universidade Federal do Rio Grande do Sul – Programa de Pós-Graduação em Ciências da Saúde: Cardiologia e Ciências Cardiovasculares, Porto Alegre, RS – Brazil; 2 Porto Alegre RS Brazil Hospital de Clínicas de Porto Alegre – Ambulatório de Cirurgia Vascular, Porto Alegre, RS – Brazil

**Keywords:** Physical Activity, Peripheral Arterial Disease, Acceleration, Velocity, Walking Speed, Exercise

Physical activity plays a key role in improving functional capacity and cardiovascular function in peripheral arterial disease (PAD).^1^ There is a direct relationship between the improvement in peak oxygen consumption (VO_2_peak) and the reduction in the risk of mortality; in addition, improving symptoms related to intermittent claudication provides a better quality of life (QoL).^2,3^ However, the assessment of the level of physical activity is often carried out in transversal studies, showing that a higher level of physical activity is correlated with a greater functional capacity, for example. However, the studies do not consider exposure over a given period, leaving open whether there are changes in cardiovascular risk parameters and physical activity levels in these individuals after a follow-up. This is what Cucato et al.,^4^ analyzed in this edition of the *Arquivos Brasileiros de Cardiologia*.

Initially, we will bring the methodological study issues, which began in 2015 and included, in the first phase, 268 patients. After 2 years of follow-up, 72 patients were reassessed in the second phase. Different cardiovascular risk parameters and physical activity levels were evaluated using a GT3X+ triaxial accelerometer (Actigraph, Pensacola, FL, USA). Here it is important to highlight the first positive point, which was to use an accelerometer to control physical activity levels, since many studies use a questionnaire to control the level of physical activity.^5^ All patients were instructed to use the accelerometer for 7 consecutive days, removing it only to sleep or shower. The device was fixed to the right side of the hip, and for analysis, a minimum of 10 hours of daily physical activity recording was required. Those who had at least 4 days of activity, 3 weekdays and 1 weekend day were considered valid. We highly value this part, as it is a simple method that provides us with extremely high-quality information.

Among the different parameters of cardiovascular risk, we can highlight blood pressure, cardiac autonomic modulation and arterial stiffness. While the functional capacity was evaluated through the 6-minute walk test. Despite being an extremely safe, effective and reproducible test, it has limitations, and here is a suggestion for future studies to evaluate VO_2_peak and its variables through a specific test using the Gardner protocol.^6–8^

Regarding the results, we can highlight that the patients reduced their total physical activity levels during these 2 years (2.257 ± 774.5 min/week pre versus 2.041 ± 676.2 min/week post, p = 0.001). Something important and worrying is that the ankle-brachial index (ABI) was also significantly reduced after the two-year follow-up (0.62 ± 0.20 pre versus 0.54 ± 0.20, p = 0.003). Why is this result worrying? Some studies show that the ABI is a prognostic marker in individuals with PAD; combining this with a reduction in physical activity levels, we will have an alarming scenario for this population. In the present study, QoL was not evaluated; however, in a worsening scenario of different parameters, we can speculate that these individuals probably worsened their QoL. From the perspective of cardiovascular parameters, we will highlight the worsening of heart rate variability measured by the standard deviation of RR intervals (p < 0.001).

One of the points that draws much attention is that it was not mentioned whether the individuals in the baseline participated in any physical training program since Dr. Cucato's research group is the reference in Brazil and one of the references in the world on PAD rehabilitation.^9^ Often, when individuals participate in a rehabilitation program, they increase their daily physical activity levels. However, at the end of the program, the tendency is to reduce their physical activity levels.^10^ The article's message is very clear, and it makes us think about the importance of monitoring these individuals over time. PAD ends up being an underdiagnosed disease; that is, individuals have symptoms but often do not care, reaching a critical level that generates several negative consequences, such as reduced functional capacity, worsening of QOL and worsening of cardiovascular parameters. New studies must be designed a priori for a long follow-up of these individuals so that we will have more information about the evolution of the disease and what benefits a higher level of physical activity can provide.
